# Inter-plane artifact suppression in tomosynthesis using 3D CT image data

**DOI:** 10.1186/1475-925X-10-106

**Published:** 2011-12-10

**Authors:** Jae G Kim, Seung O Jin, Min H Cho, Soo Y Lee

**Affiliations:** 1 Department of Biomedical Engineering, Kyung Hee University, Korea; 2 Korea Electrotechnology Research Institute, Korea; 3 Department of Electronic Engineering, Hanyang University, Korea

## Abstract

**Background:**

Despite its superb lateral resolution, flat-panel-detector (FPD) based tomosynthesis suffers from low contrast and inter-plane artifacts caused by incomplete cancellation of the projection components stemming from outside the focal plane. The incomplete cancellation of the projection components, mostly due to the limited scan angle in the conventional tomosynthesis scan geometry, often makes the image contrast too low to differentiate the malignant tissues from the background tissues with confidence.

**Methods:**

In this paper, we propose a new method to suppress the inter-plane artifacts in FPD-based tomosynthesis. If 3D whole volume CT images are available before the tomosynthesis scan, the CT image data can be incorporated into the tomosynthesis image reconstruction to suppress the inter-plane artifacts, hence, improving the image contrast. In the proposed technique, the projection components stemming from outside the region-of-interest (ROI) are subtracted from the measured tomosynthesis projection data to suppress the inter-plane artifacts. The projection components stemming from outside the ROI are calculated from the 3D whole volume CT images which usually have lower lateral resolution than the tomosynthesis images. The tomosynthesis images are reconstructed from the subtracted projection data which account for the x-ray attenuation through the ROI. After verifying the proposed method by simulation, we have performed both CT scan and tomosynthesis scan on a phantom and a sacrificed rat using a FPD-based micro-CT.

**Results:**

We have measured contrast-to-noise ratio (CNR) from the tomosynthesis images which is an indicator of the residual inter-plane artifacts on the focal-plane image. In both cases of the simulation and experimental imaging studies of the contrast evaluating phantom, CNRs have been significantly improved by the proposed method. In the rat imaging also, we have observed better visual contrast from the tomosynthesis images reconstructed by the proposed method.

**Conclusions:**

The proposed tomosynthesis technique can improve image contrast with aids of 3D whole volume CT images. Even though local tomosynthesis needs extra 3D CT scanning, it may find clinical applications in special situations in which extra 3D CT scan is already available or allowed.

## Background

Tomosynthesis is now gaining its important roles in clinical diagnosis owing to its dual features of CT and radiography. Tomosynthesis can be incorporated into many kinds of x-ray imaging systems that are equipped with a flat panel detector (FPD) as an image acquisition device. Such FPD-based x-ray imaging systems include C-arm imaging systems, mammography systems, and some DR systems. As compared to image intensifiers, FPDs have no spatial distortion in image acquisition, which is essential for accurate image reconstruction in tomosynthesis. Unlike CT imaging where axial resolution is of main concern, tomosynthesis aims at taking high lateral-resolution images for which fine pixel-pitch FPDs are well fitted [1,2]. Aside from the superior lateral resolution, tomosynthesis has great clinical potential because of its lower x-ray dose as compared to CT [3,4].

Due to the limited scan angle of tomosynthesis, usually less than 60°, the off-focal plane components are not completely cancelled out in the tomosynthesis image reconstruction [5-7]. The partial cancellation of the off-focal components limits the depth resolving power of thomosynthesis and it makes the slice profile in the depth direction extended far away from the focal plane [1]. The extended slice profile often makes inter-plane artifacts particularly when high intensity structures exist in off-focal planes [8]. The inter-plane artifacts, also called ghost artifacts, could mislead the diagnosis severely compromising the clinical utility of tomosynthesis.

With a cone-beam CT equipped with a FPD as an image acquisition device, tomosynthesis scan can be easily performed by simply limiting the scan angle [9,10]. By taking high-resolution projection images at multiple viewing angles in the CT scan geometry and combining the multiple projection images in a way that those are coherently added on a focal plane, tomosynthesis images can be made in the framework of cone-beam CT. When repetitive follow-up imaging of a region of interest (ROI) is necessary after localizing the ROI by CT scan as in the case of radio-therapy and dental implanting, tomosynthesis would be suitable for the follow-up imaging because of its faster scan time and lower x-ray dose than CT's. In such a situation, the CT image data can be incorporated into the tomosynthesis image reconstruction to remove the inter-plane artifacts. Once the off-focal components, which account for the projection components outside the ROI, have been computed from the 3D CT image data, the off-focal components can be subtracted from the tomosynthesis projection data for removal of the inter-plane artifacts. We call this technique local tomosynthesis in that the projection components stemming from a local ROI are only involved in the tomosynthesis image reconstruction. We have verified the local tomosynthesis concept through simulations and real experiments with a micro-CT, and we present experimental results of the local tomosynthesis in comparison with the ones obtained from the conventional tomosynthesis.

## Methods

### Calculation of the projection components stemming from outside the region of interest

**Figure 1:**
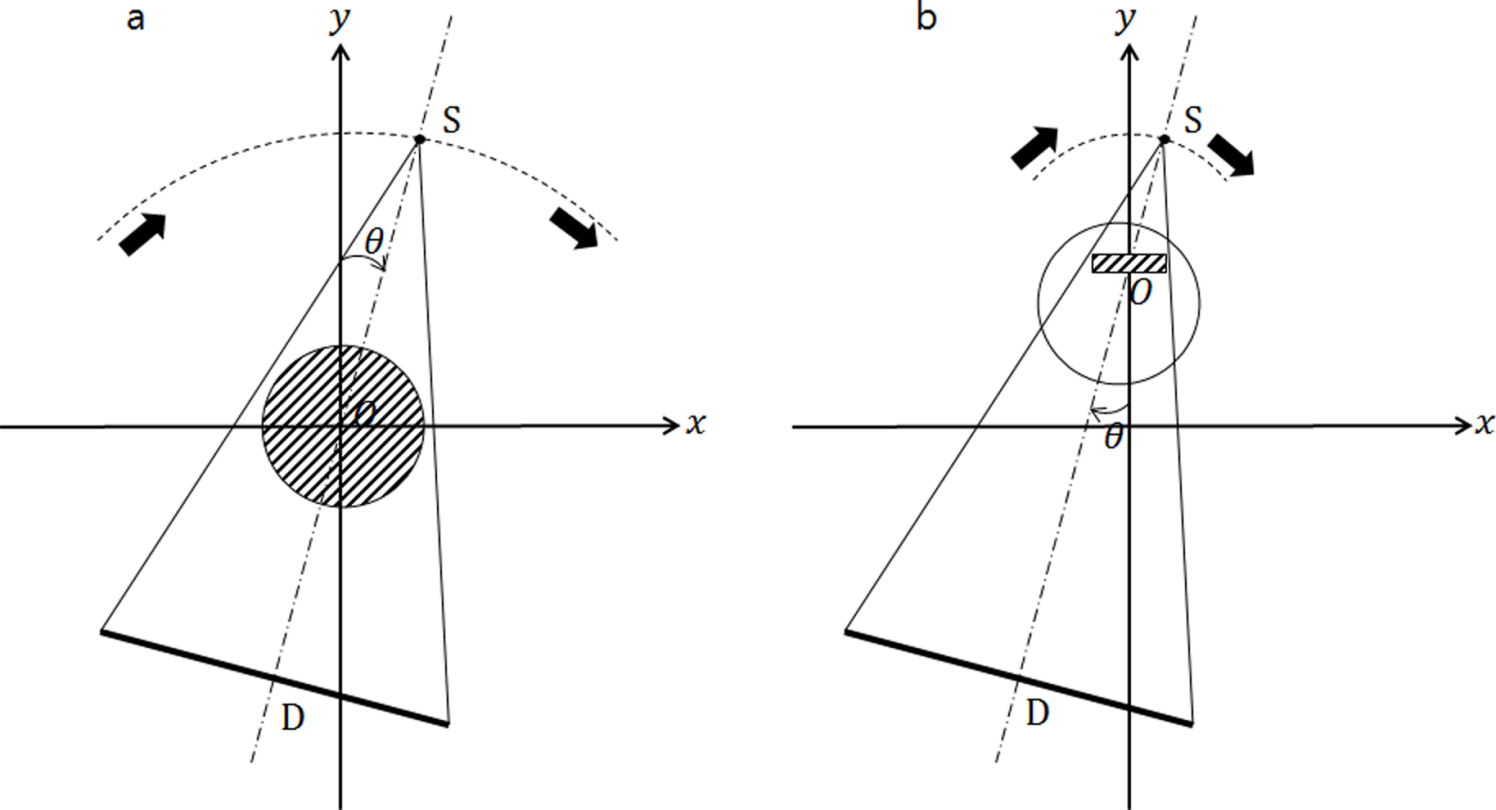
**Scan geometries of the CT and the tomosynthesis**. (a) The CT scan geometry with small magnification. (b) The tomosynthesis scan geometry with big magnification. *S *and *D *represent the x-ray source and detector, respectively, and *O *represents the center of rotation of the scan.

For local tomosynthesis of a ROI, we use 3D CT image data obtained prior to the tomosynthesis scan with the imaging object positioned at the same place at both scans, that is, one for the CT scan and the other for the tomosynthesis scan. We assume here that the 3D CT scan covers the whole imaging volume whilst the tomosynthesis scan may cover only a small ROI. Figure 1 dictates the scan geometry of the 3D CT scan and the tomosynthesis scan. In Figure 1a, the 3D CT scan is performed with the x-ray source and detector pair rotating 360 degrees. The center of rotation of the x-ray source **S **and detector **D **is denoted as **O**. In Figure 1b, the tomosynthesis scan is performed with the scan angle θ varying from Θ to Θ. Θ mostly ranges at 20°~30° in most of tomosynthesis applications. The centers of rotation in the CT and tomosynthesis may not be necessarily the same. In the tomosynthesis scan, the center of rotation **O **may be moved toward the x-ray source to get higher magnification ratio of the projection data, hence, higher lateral resolution if the scanning mechanism permits such displacement of the x-ray source and detector pair as introduced in our previous works [11-13]. The magnification ratio of the projection data is determined by *SDD*/*SOD *where *SDD *and *SOD *are the source-to-detector and source-to-object distances, respectively. Neglecting the focal spot size effect of the x-ray target, we can improve the spatial resolution of the projection data by increasing the magnification ratio.

**Figure 2:**
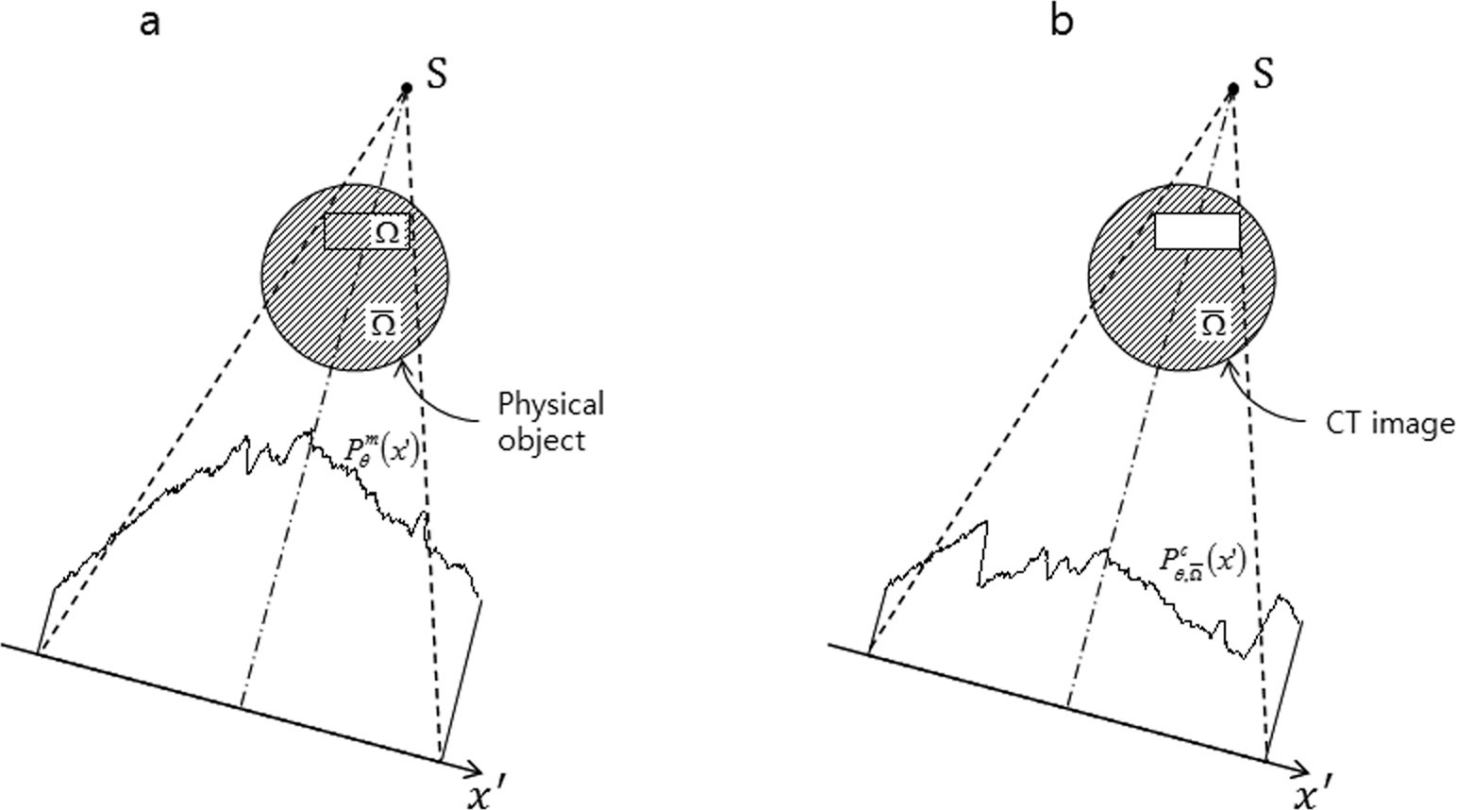
**The two projection data for image reconstruction**. (a) The projection data acquired from the tomosynthesis scan. (b) The projection data calculated from the CT image which stems from outside the region of interest.

In Figure 2, the ROI Ω is located off center for the sake of generality of local tomosynthesis. We denote outside the ROI as$\bar{\Omega }$. The projection data measured from the physical imaging object at scan angle θ and detector position *x*' is denoted as $P_{\theta }^{m}\left(x^{\prime }\right)$ in Figure 2a. In Figure 2b, we show the CT image for calculating the projection component stemming from outside the ROI. The calculated projection data$P_{\theta ,\bar{\Omega }}^{c}\left(x^{\prime }\right)$, which takes account of only outside the ROI, can be calculated as follows:

(1) $$P_{\theta ,\bar{\Omega }}^{c}\left(x^{\prime }\right)=\underset{L\subset \bar{\Omega }}{\int }\mu_{c}\left(x,y\right)dl.$$

The measured projection data passing through the physical imaging object is given as follows:

(2) $$P_{\theta }^{m}\left(x^{\prime }\right)=\underset{L\subset \left(\Omega +\bar{\Omega }\right)}{\int }\mu_{p}\left(x,y\right)dl.$$

In the above equations, *L *is the x-ray path, *μ_c _*is the x-ray attenuation coefficient represented by the CT image and *μ_p _*is the physical x-ray attenuation coefficient. *μ_c _*and *μ_p _*may differ from each other because of many physical factors as will be described later. If we assume *μ_c _*and *μ_p _*be the same, then, we can separate the projection components, one from the ROI and the other from outside the ROI, in the measured projection data. That is, the projection components stemming from the ROI is given as,

(3) $$P_{\theta ,\Omega }^{c}\left(x^{\prime }\right)=P_{\theta }^{m}\left(x^{\prime }\right)-P_{\theta ,\bar{\Omega }}^{c}\left(x^{\prime }\right).$$

The measured projection data would have higher spatial resolution, owing to the high magnification ratio at the tomosynthesis scan, than the projection data computed from the CT image. Therefore, low-resolution and high-resolution projection components may be mixed together in$P_{\theta ,\Omega }\left(x^{\prime }\right)$.

As stated above, the attenuation coefficients represented by the CT images may not match well with the physical attenuation coefficients. Firstly, the beam hardening effects may differ in the CT scan and the tomosynthesis scan since the x-ray paths differ in both scans. Secondly, the x-ray CT images always have a certain level of streak artifacts due to the limited number of samplings in the angular scan direction. Due to the streak artifacts, the pixels in the background air region always have non-zero attenuation coefficients. There will be many other factors, such as finite pixel size of the CT images, finite detector element size, and nonlinear response of the detector, that account for the mismatches between the two attenuation coefficients. Here, we simply assume that the projection data computed from the CT image can be approximated by scaling and biasing the measured projection data, that is:

(4) $$P_{\theta }^{c}\left(x^{\prime }\right)=\underset{L\subset \left(\Omega +\bar{\Omega }\right)}{\int }\mu_{c}\left(x,y\right)dl=aP_{\theta }^{m}\left(x^{\prime }\right)+b.$$

Then, we find the pair $\left(\hat{a},\hat{b}\right)$ that best matches the two projection data in terms of mean square error:

(5) $$(\hat{a},\hat{b})=\underset{\left(a,b\right)}{\arg\min}{\left\|P_{\theta }^{c}(x^{\prime })-aP_{\theta }^{m}(x^{\prime })-b\right\|}^{2}.$$

Once $\left(\hat{a},\hat{b}\right)$ found, we correct the computed projection data as follows:

(6) $$P_{\theta ,\bar{\Omega }}^{c}\left(x^{\prime }\right)\leftarrow \frac{1}{\hat{a}}\left(P_{\theta ,\bar{\Omega }}^{c}\left(x^{\prime }\right)-\hat{b}\right).$$

### Tomosynthesis image reconstruction

After computing the projection components stemming from the ROI, $P_{\theta ,\bar{\Omega }}^{c}\left(x^{\prime }\right)$, we reconstruct tomosynthesis images using the maximum likelihood (ML)-convex method. The ML-convex method is one of the statistical reconstruction methods that take account of measurement statistics and noise model. Assuming that the incident and transmitted x-ray intensities follow Poisson statistics and the measured intensities at the detector pixels are mutually independent to one another, the pixel value estimated by the ML-convex method is given by [7]:

(7) $${\overset{⌢}{x}}_{j}^{k+1}={\overset{⌢}{x}}_{j}^{k}+\lambda \frac{{\overset{⌢}{x}}_{j}^{k}\overset{N}{\underset{n=1}{\sum }}\overset{I}{\underset{i=1}{\sum }}a_{ij,n}\left(I_{0,n}e^{-{\left\langle a,x\right\rangle }_{i,n}}-I_{i,n}\right)}{\overset{N}{\underset{n=1}{\sum }}\overset{I}{\underset{i=1}{\sum }}a_{ij,n}{\left\langle a,x\right\rangle }_{i,n}I_{0,n}e^{-{\left\langle a,x\right\rangle }_{i,n}}}$$

where *a_ij,n _*is the path length of the ray at the *j*-th pixel that reaches the *i*-th detector element in the *n*-th projection view, *k *is the iteration number, *I_i,n _*is the measured projection data at the *i*-th detector element in the *n*-th projection view, 〈*a*, *x*〉*_i,n _*is the line integral of the estimated attenuation coefficients along the x-ray path that hits the *i*-th detector element in the *n*-th projection view, and *I*_0*,n *_is the incident x-ray beam intensity in the *n*-th view. *N *and *I *are the total number of projection views and the number of detector elements, respectively. In the reconstruction of tomosynthesis images using the ML-convex method, we use uniform images as the initial guess of the reconstruction and we set the step size λ to 1. We stop the iteration evaluating the image quality by visual inspection.

### Image quality evaluation

The main advantage of the proposed method over the conventional tomosynthesis method is the improvement in contrast-to-noise ratio owing to the reduction of inter-plane artifacts. If the background in the imaging object is perfectly uniform, the subtraction effects in the proposed method would be minimal since there is no inter-plane artifact stemming from the uniform background. But, in heterogeneous tissue imaging, the background is not uniform and it could make quite noticeable inter-plane artifacts. The inter-plane artifacts may be considered as noise that hampers readability of the reconstructed images. For the quantitative evaluation of inter-plane artifact suppression, we consider a figure of merit, contrast-to-noise ratio (CNR) [7]. CNR takes account of contrast, between the feature of interest and the background, and noise power. In tomosynthesis, CNR is affected by the generic noises, stemming from the x-ray photon noise and the electronic noise from the detector, and the inter-plane artifacts. We calculate CNR at the feature of interest on its focal plane. The CNR is defined by:

(8) $$CNR=\frac{I_{S}-I_{B}}{\sigma_{BG}}$$

where *I_s _*is the mean pixel intensity at the feature region, *I_B _*is the mean pixel intensity at the nearby background and *σ_BG _*is the standard deviation of the pixel intensity at the nearby background.

### Experimental set up

**Figure 3:**
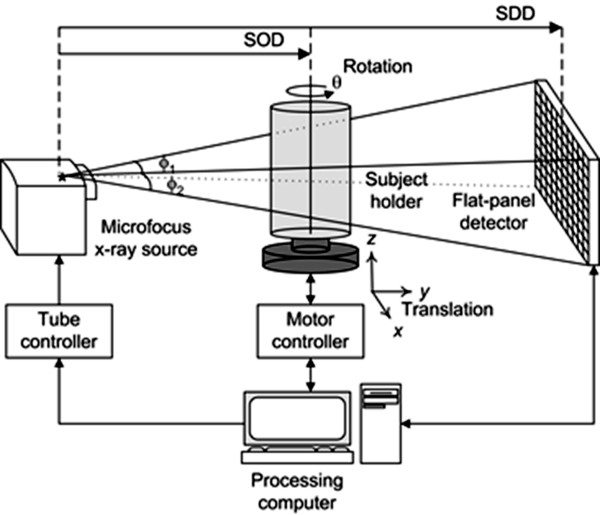
**The micro-CT system used for the CT and tomosynthesis scans**. The magnification of the projection image on the flat-panel detector is controlled by changing the source to object distance (SOD).

For the CT and tomosynthesis scans, we have used a lab-built micro-CT system whose schematic diagram is shown in Figure 3[14]. The micro-CT system consists of a micro-focus x-ray source, a CMOS FPD and a rotating object holder in between them. The micro-focus x-ray source and the FPD are fixed on a bench whilst the rotating object holder can be displaced along the line connecting the x-ray source and the FPD by the ball-screw driven sliding mechanism. Therefore, the source-to-object distance, shortly *SOD*, can be adjusted within a given range with the source-to-detector distance, *SDD*, fixed to a given value. The magnification ratio of the projection image, given by *SDD*/*SOD*, can be controlled by adjusting *SOD*. The micro-focus x-ray source (L8121-01, Hamamatsu, Japan) has a fixed tungsten anode with the target face angle of 25° against the electron beam, and it has a 200 μm-thick beryllium exit window. The span angle of the x-ray cone beam emitted from the exit window is about 43°. The x-ray source has a variable focal spot size that ranges from 5 μm to 50 μm depending on the applied tube power. The micro-focus x-ray source has been operated in a continuous mode with a 1 mm thick Al filter. A commercially available flat-panel detector (C7942SK-02, Hamamatsu, Japan) has been used as a 2D digital x-ray imager in the micro-CT system. The flat-panel detector consists of a 2240 × 2240 active matrix of transistors and photodiodes with a pixel pitch of 50 μm and a CsI:Tl columnar structured scintillator.

### Simulations and imaging experiments

**Figure 4:**
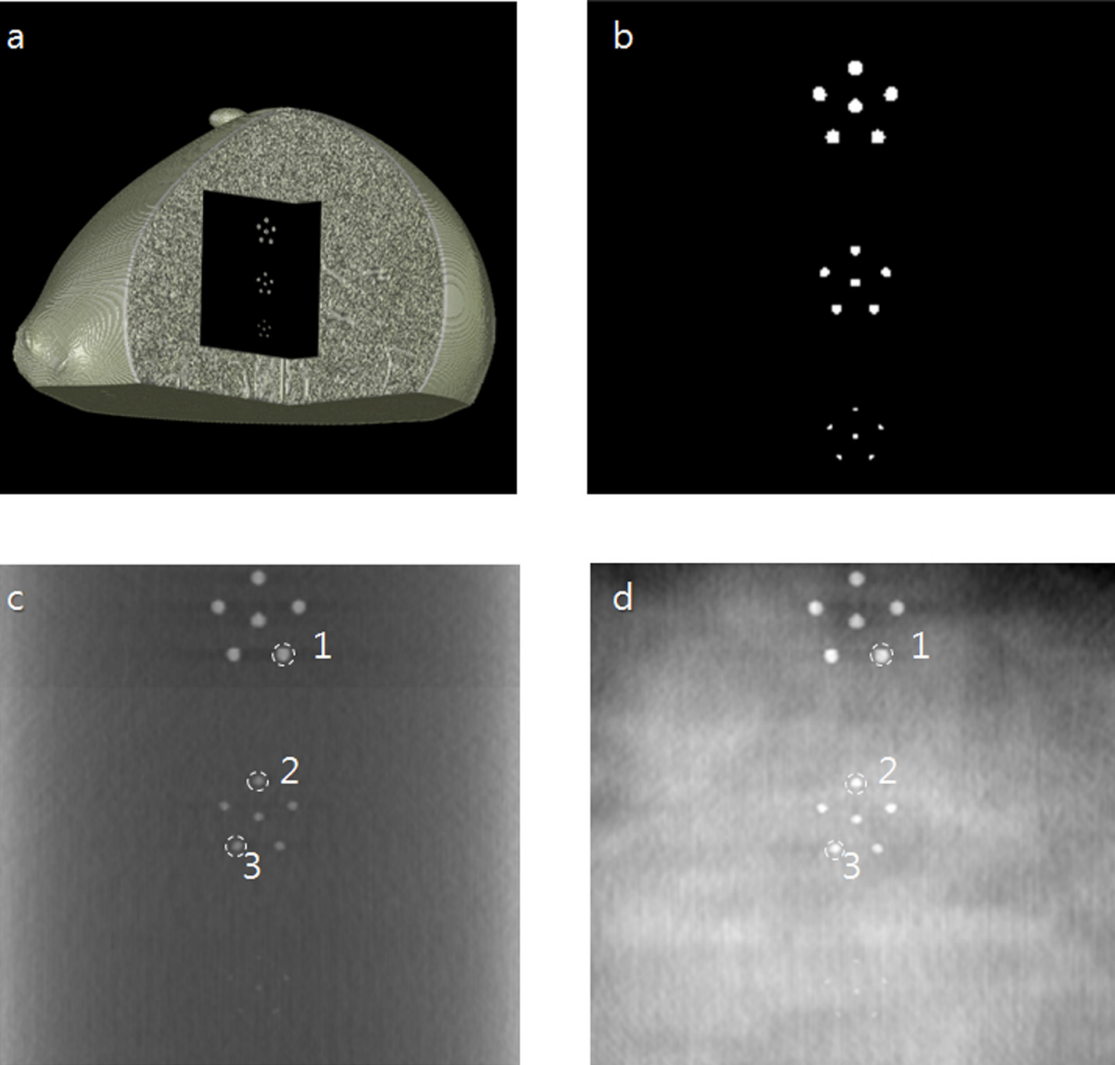
**The breast-mimicking phantom for simulation and its simulated tomosynthesis images**. (a) The 3D breast-mimicking phantom for the simulation. (b) The high intensity feature patterns located in the middle of the 3D breast-mimicking phantom. (c) The simulated local tomosynthesis image obtained with the proposed method. (d) The simulated conventional tomosynthesis image. The numbers are the places where CNRs are to be evaluated.

We first verify the proposed method by reconstructing tomosynthesis images from the simulated projection data taken from a breast-mimicking 3D phantom shown in Figure 4a. The phantom has the matrix size of 800 × 800 × 650 with the physical voxel size of 0.084 × 0.084 × 0.084 mm^3^. The phantom has the featured layer, shown in Figure 4b, in the middle of it for the quantification of CNR performance. The featured layer has high-intensity spheres against uniform background. The diameters of three types of spheres are 0.50 mm, 0.34 mm and 0.17 mm. The physical size of the phantom is 55 × 55 × 37 mm^3 ^when measured by its longest axes. The phantom consists of 3D voxels mimicking fibro-glandular tissues, ductal tissues and fatty adipose tissues [15].

**Figure 5:**
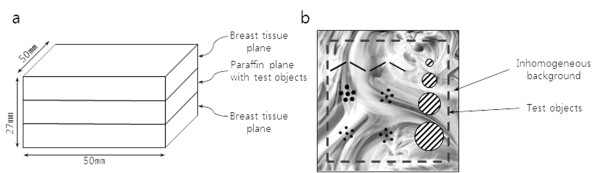
**The breast-mimicking phantom used for the experimental scans**. (a) The phantom consists of three layers. The top and bottom layers are simulating inhomogeneous breast tissues. (b) The middle layer having high-intensity feature patterns against the homogeneous background.

To experimentally verify the proposed method, we first take images of a phantom shown in Figure 5. The phantom consists of two breast-mimicking layers the size of 50 × 50 × 10 mm^3 ^and a featured layer the size of 50 × 50 × 7 mm^3 ^stacked in parallel as shown in Figure 5a. We made the breast-mimicking layers by cutting off square blocks from commercial breast-mimicking phantoms (BR3D(Model 020), CIRS, U.S.A.). The breast-mimicking layers simulate adipose and glandular tissues of a human breast. The middle layer is made of uniform paraffin with high-attenuation feature patterns in it. Figure 5b shows the feature patterns on top of the inhomogeneous layer. We also take images of an adult rat *post mortem *to see the performance of the proposed method in small animal imaging. We have performed all the animal experiments under the regulations of the Institutional Review Committee at Kyung Hee University.

## Results

### Simulation results

We numerically calculated the projection data from the simulation phantom shown in Figure 4a with the magnification ratios of 1:3.9 for the tomosynthesis scan and 1:1.3 for the CT scan. The numbers of views were 360 and 31 for the CT scan and tomosynthesis scan, respectively, and the scan angles were 360° and 60° for the CT scan and tomosynthesis scan, respectively. The detector matrix size was assumed to be 1216 × 1216 at the calculation of the CT projection data. Using the FDK algorithm [16], we have reconstructed 256 × 256 × 256 3D CT images of the phantom from the projection data covering the entire phantom. From the 3D CT images, we calculated the tomosynthesis projection data stemming from outside the ROI, that is, from$\bar{\Omega }$, of the CT images. We set the ROI around the featured layer. From the 3D phantom, we have also calculated the tomosynthesis projection data stemming from $\Omega +\bar{\Omega }$ simulating the measured projection data. We have subtracted the two projection data from one another to make the projection data stemming from Ω. With the subtracted projection data stemming from Ω, we have reconstructed tomosynthesis images using the ML-convex algorithm. One of the local tomosynthesis images of the featured layer is shown in Figure 4c. For the sake of comparison, we have also reconstructed conventional tomosynthesis images from the projection data stemming from the entire region, $\Omega +\bar{\Omega }$, and we have shown it in Figure 4d. As can be noticed from Figure 4d, the image obtained by the conventional tomosynthesis method has stronger inter-plane artifacts coming from off-focal planes. We have calculated CNRs at the numbered feature patterns in Figure 4 and we have shown them in Table 1. In calculating the standard deviation for CNR, we took account of 7 × 7 pixels in the background around the feature patterns. As can be noticed from Table 1, the CNR improvement varies depending on the degree of tissue inhomogeneity around the feature patterns.

**Table 1:** CNRs measured at the three feature patterns in the simulated images

CNR		
**ROI**	**Conventional tomosynthesis**	**Local tomosynthesis**

1	10.7	11.5

2	8.2	30.1

3	16.2	20.7

**Figure 6:**
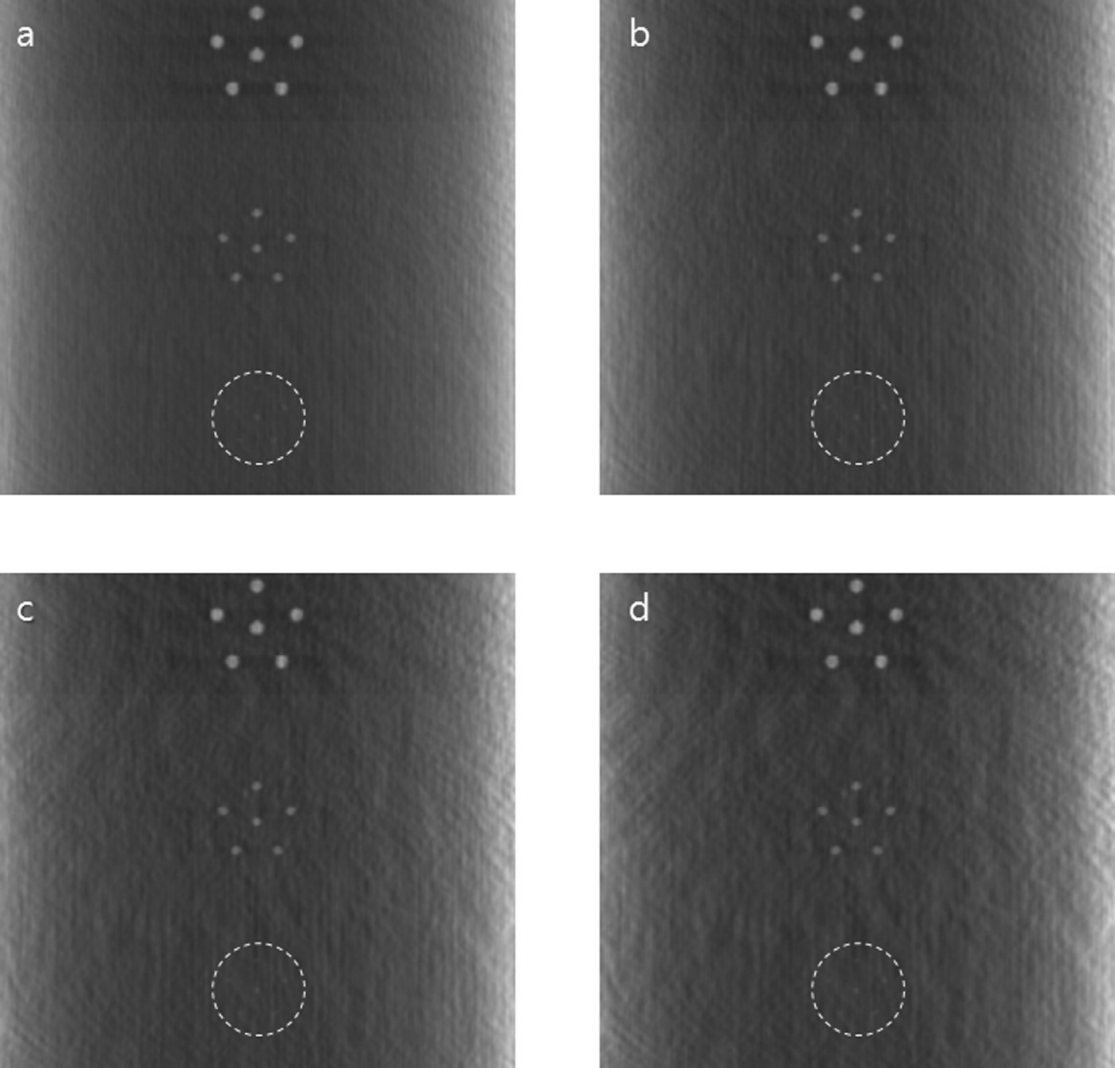
**The misalignment effects in simulated local tomosynthesis images**. The images have been obtained with misregistration between the CT and tomosynthesis scan geometry. The misregistrations are made by shifting the CT projection data in the horizontal direction by multiple pixel widths. (a) 0-pixel shift. (b) 2-pixel shift. (c) 4-pixel shift. (d) 8-pixel-shift.

To observe the effects of scan geometry misregistration between the CT and tomosynthesis scans, we intentionally shifted the CT projection data in the horizontal direction by multiple pixel widths before the subtraction of the two projection data. Figure 6 shows the local tomosynthesis images obtained with the misregistered CT projection data. The degrees of misregistration are 2-, 4-, 6-, and 8-pixel widths, respectively. As can be seen from Figure 6, the misregistration effects are not significant in 2- and 4-pixel shift cases. However, we can see significant increase of inter-plane artifacts along with loss of visibility of the small feature pattern (circled region in the figure) in the 8-pixel shift case.

### Experimental results

**Figure 7:**
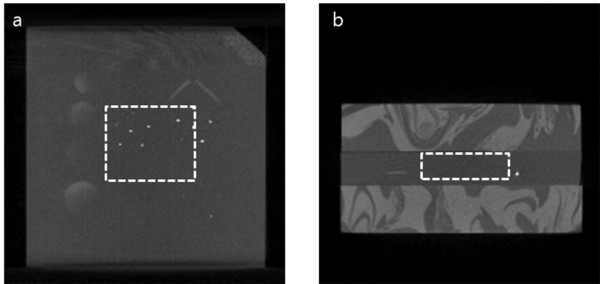
**The 3D CT images of the breast-mimicking phantom**. (a) One example image in the coronal view. (b) One example image in the axial view. The white rectangle is the ROI for the local tomosynthesis.

We have first taken 3D CT images of the phantom shown in Figure 5 with the matrix size of 256 × 256 × 256, and we have shown a coronal-view image and an axial-view image in Figure 7a and 7b, respectively. In the CT scan, the x-ray tube voltage and tube current were 54 kVp and 0.5 mA, respectively, the number of views was 360, and the magnification ratio of the CT scan was 1:1.3. We have reconstructed the 3D CT images using the FDK algorithm with the isotropic voxel size of 340 × 340 × 340 μm^3^.

**Figure 8:**
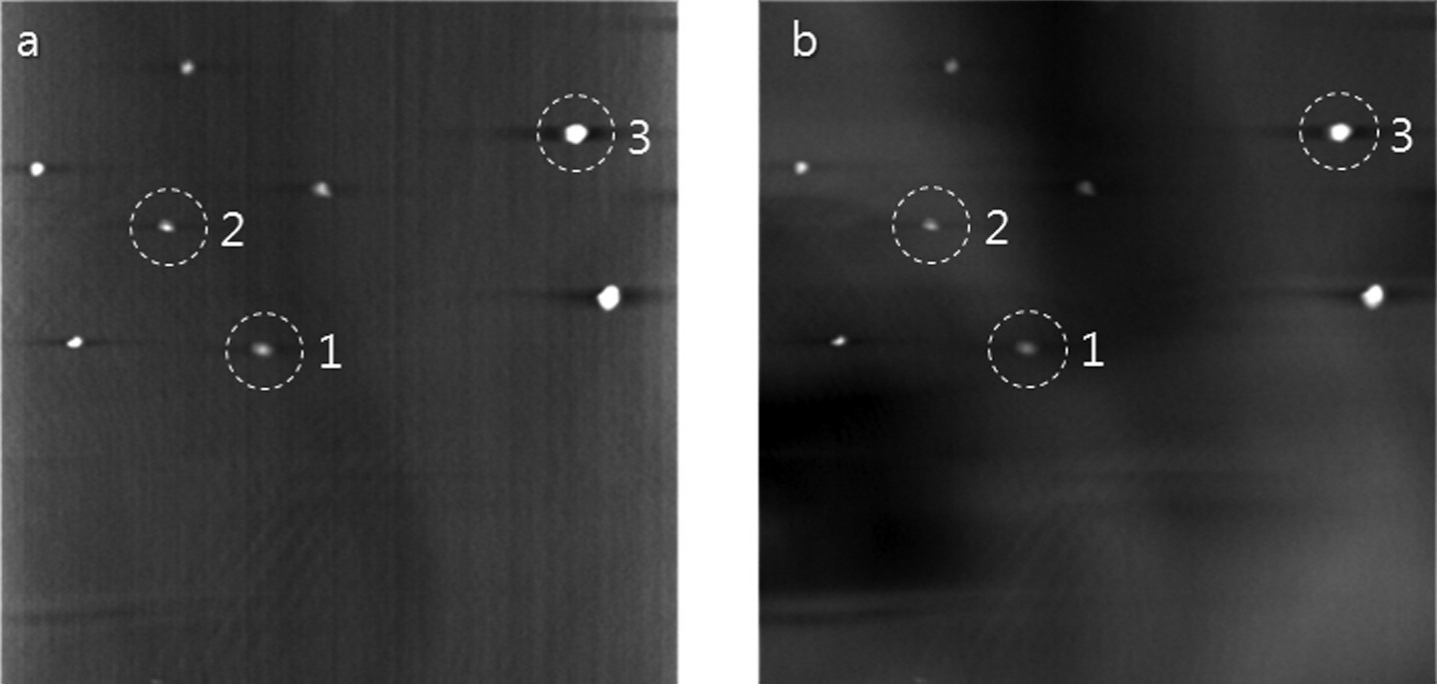
**The experimental tomosynthesis images of the phantom**. (a) The image obtained by the proposed method. (b) The image obtained by the conventional method. The numbers are the places where CNRs are to be evaluated.

We set the ROI for the local tomosynthesis around the dot feature patterns as depicted by the white rectangles in Figure 7. Maintaining the same tube voltage and tube current, we performed the tomosynthesis scan with the magnification ratio of 1:3.9. At the tomosynthesis scan, the number of views was 31 with the incremental angular step of 2°. After obtaining the projection data stemming from Ω, we have reconstructed local tomosynthesis images using the ML convex algorithm and we have shown one of them in Figure 8a. In the iterative reconstruction, the number of iteration steps was seven. The focal plane of the tomosynthesis image reconstruction was the same lateral plane of the CT image shown in Figure 7a. For the sake of comparison, we have also reconstructed conventional tomosynthesis images using the measured projection data, stemming from $\Omega +\bar{\Omega }$, and we have shown the image on the same focal plane in Figure 8b. The number of iteration steps was set to seven in this case too. As can be noticed from Figure 8, the conventional tomosynthesis image has much bigger inter-plane artifacts arising from the inhomogeneous off-focal planes. We calculated CNRs at the three feature patterns circled in Figure 8 for two types of images, and we summarized them in Table 2. As can be noticed from Table 2, the local tomosynthesis gives higher CNRs than the conventional tomosynthesis owing to the suppression of the inter-plane artifacts.

**Table 2:** CNRs measured at the three feature patterns in the experimental images

CNR		
**ROI**	**Conventional tomosynthesis**	**Local tomosynthesis**

1	85.1	105.0

2	213.9	767.0

3	72.9	148.1

**Figure 9:**
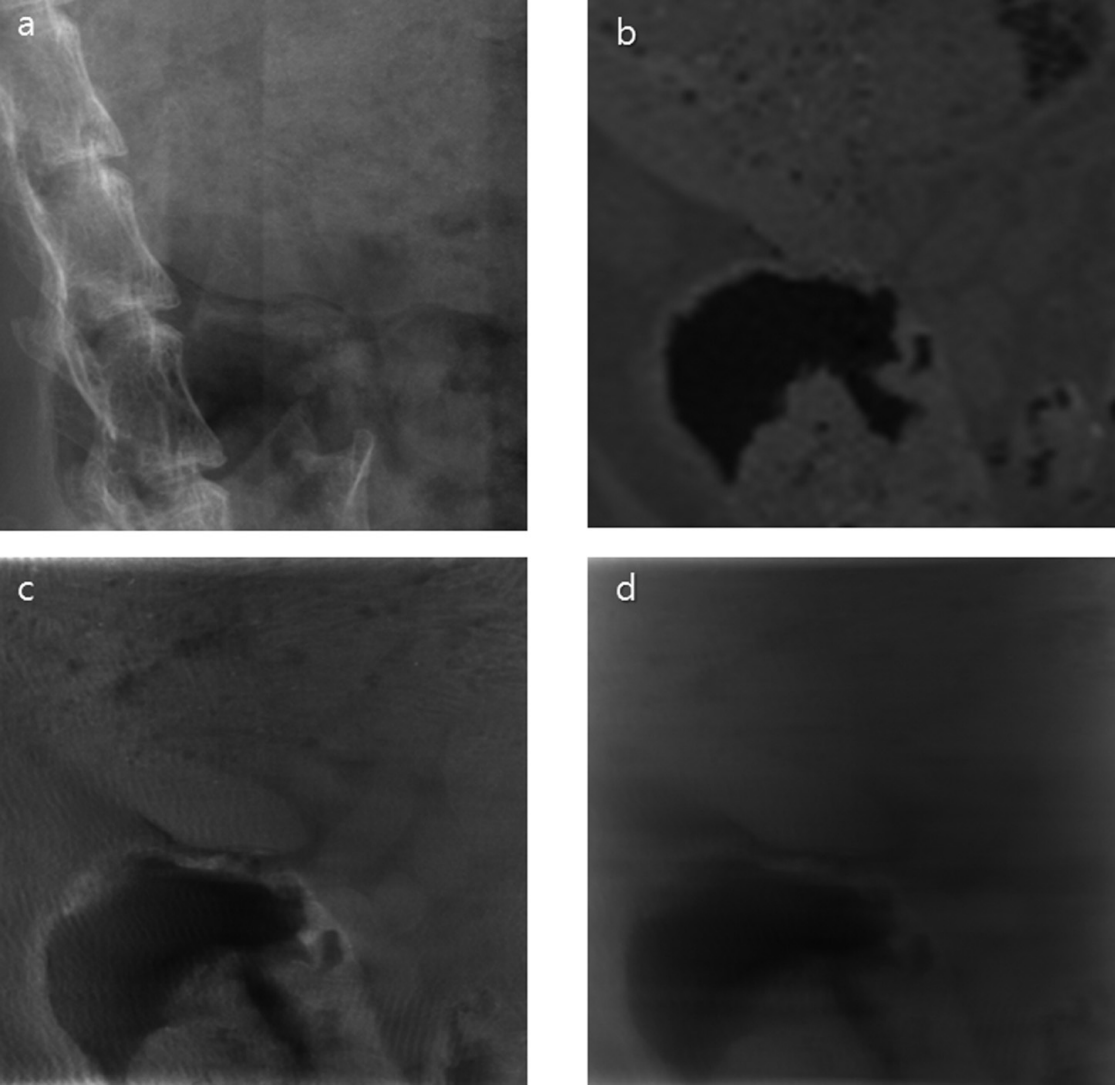
**Post-mortem rat imaging results**. (a) Projection image (b) An axial view of 3D CT images. The rectangular box indicates the ROI. (c) A tomosynthesis image in the ROI obtained with the proposed method. (d) A tomosynthesis image in the ROI obtained with the conventional tomosynthesis method.

**Figure 10:**
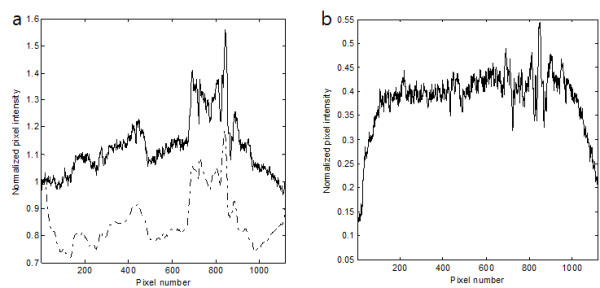
**Examples of the experimental projection data**. (a) A projection data taken from the rat imaging. The solid line represents the measured tomosynthesis projection data and the dashed line represents the projection data computed from the CT images. The computed projection data has been obtained from outside the rectangular ROI shown in Fig. 9b. (b) The subtracted projection data which stems from the ROI.

To test the proposed method for animal imaging, we have taken 3D CT images of an adult rat *post mortem *using the micro-CT. The x-ray tube voltage and tube current were 65 kVp and 0.34 mA, respectively, the number of views was 900, and the magnification ratio of the CT scan was 1:1.3. We have reconstructed the 3D CT images with the matrix size of 256 × 256 × 256 and the isotropic voxel size of 340 × 340 × 340 μm^3^. One of the projection images and one of the coronal view CT images are shown in Figure 9a and 9b, respectively. Maintaining the same tube voltage and tube current, we performed the tomosynthesis scan with the magnification ratio of 1:3.9. At the tomosynthesis scan, the number of views was 31 with the incremental angular step of 2°. In Figure 10a, we have shown examples of the two projection data, one from the real tomosynthesis scan stemming from $\Omega +\bar{\Omega }$ and the other from the CT images stemming from $\bar{\Omega }$. After obtaining the projection data stemming from Ω as shown in Figure 10b, we have reconstructed local tomosynthesis images using the ML convex algorithm, and we have shown one of them in Figure 9c. We have also reconstructed conventional tomosynthesis images using the measured projection data, stemming from $\Omega +\bar{\Omega }$, and shown the image on the same focal plane in Figure 9d. The number of iteration steps was 7 for both tomosynthesis image reconstructions. As can be noticed from the two tomosynthesis images, the local tomosynthesis image shows higher contrast than the conventional tomosynthesis image.

## Discussion

The contrast improvement in the proposed local tomosynthesis is largely due to the suppression of inter-plane artifacts stemming from the off-focal planes. Therefore, the degree of contrast improvement will be spatially variant depending on the tissue inhomogeneity in off-focal planes. The contrast enhancement will be maximized when the two projection data, one from the CT scan and the other from the tomosynthesis scan, are best registered in terms of scan geometry and x-ray attenuation. If the two scan geometries are not registered perfectly in the subtraction of the two projection data, the cancellation of the off-focal components will not be complete and residual inter-plane artifacts may persist. We have performed the experiments with a small-scale micro-CT in which the scan geometry can be rather precisely controlled by precision mechanism. But, in a large-scale human imaging device, changing the scan geometry in precision will be a technical challenge. The two projection data may not be registered well due to different x-ray attenuation along the different projection lines in the CT and tomosynthesis scans. The polychromatic x-ray attenuation is never linear due to beam hardening and scattering of the x-ray beam. So, the two projection data obtained with different magnification ratios may not be registered well in the subtraction. But, the experimental results obtained under different magnification settings in this study have shown that inter-plane artifacts in tomosynthesis can be suppressed to some degree by the proposed technique. The advantage of high magnification ratio imaging in tomosynthesis may be offset due to the penumbra effect caused by the finite-sized x-ray focal spot. Therefore, we have to carefully choose the magnification ratio to get optimal spatial resolution in the tomosynthesis scan.

For practical use of the proposed method, we have to address many technical issues. Firstly, we have to establish an efficient way to exactly register the two scan geometries, one for the CT scan and the other for the tomosynthesis scan. For given scan geometries for the CT and tomosynthesis scans, we have to register them in the framework of the projection data subtraction in a fast and automatic way. Since the estimation of the projection components stemming from the ROI is based on the subtraction, the local tomosynthesis images are sensitive to the subtraction errors. In the present experiments, we have manually measured the scan geometries by taking two projection images of a thin planar structure with different magnification ratios. After taking the two projection images, we found the magnification ratios of the two scans and translational/rotational mismatches between the two scans using the image registration technique. Secondly, we have to find the extent of ROI size to which we can take advantage of local tomosynthesis. If the ROI is too small as compared to the whole volume size, small minor errors in estimating the projection components from the CT images may lead to significant errors in reconstructing tomosynthesis images. In the future studies, we should perform further investigations on the effects of the aforementioned factors on the local tomosynthesis image quality.

A clear limitation of the proposed method is that we need an extra CT scan to get the 3D information about attenuation coefficient distribution. In most cases, 3D CT scanning would suffice since 3D CT images usually provide sufficient anatomical information of the imaging region. However, in the situation where extra tomosynthesis scan is allowed in addition to the 3D CT scan or vice versa, the proposed local tomosynthesis technique will find advantages over the conventional tomosynthesis technique. We think local tomosynthesis may find its clinical application particularly in dental imaging. For dental implanting, we usually obtain 3D CT images of the whole teeth. However, it is often the case that we need high-resolution lateral views of the teeth of interest or the dental implants to better position them on the mandibula or maxilla bones. Considering the fact that low dose CT imaging techniques have been widely developing [17-20], we may consider extra low dose CT scan for the local tomosynthesis in some clinical applications using a C-arm CT.

## Conclusions

The proposed local tomosynthesis technique can significantly suppress the inter-plane artifacts improving tomosynthesis image contrast with aids of 3D whole volume CT images. Even though local tomosynthesis needs extra 3D CT scanning, it may find clinical applications in special situations in which extra 3D CT scan is already available or allowed.

## Competing interests

The authors declare that they have no competing interests.

## Authors' contributions

JG carried out the implementation of the proposed idea and performed the experiments using a FPD-based micro-CT. JG also carried out the image reconstruction and image analysis and drafted the manuscript. SO advised on the implementation of the ML-convex algorithm and carried out the data analysis. MH participated in the experiment design and data analysis. SY conceived of this study, participated in its design, analysis and interpretation of the data, and helped draft and finalize the manuscript. All authors have read and approved the final manuscript.
